# Illness costs to households are a key barrier to access diagnostic and treatment services for tuberculosis in Tajikistan

**DOI:** 10.1186/1756-0500-3-340

**Published:** 2010-12-20

**Authors:** Raffael Ayé, Kaspar Wyss, Hanifa Abdualimova, Sadullo Saidaliev

**Affiliations:** 1 Swiss Tropical and Public Health Institute, Swiss Centre for International Health, Socinstr. 57, 4002 Basel, Switzerland; 2 University of Basel, Basel, Switzerland; 3 Project Sino, Rudaki prospekt proyezd 5, dom 1, Dushanbe, Tajikistan; 4 Ministry of Health and Republican Centre for Tuberculosis Control, Bukhoro Street 53, Dushanbe, Tajikistan

## Abstract

**Background:**

Tuberculosis (TB) control is based on early detection and complete treatment of infectious cases. Consequently, it is important that TB suspects and patients can readily access medical care. This qualitative study investigated determinants of access to DOTS services as identified by patients, health providers and community members in four districts in Tajikistan.

**Findings:**

Focus group discussions were conducted in order to investigate access to TB services. A conceptual framework for access to care guided the analysis. Thirteen focus group discussions involving a total of 97 informants were conducted. Content analysis of discussions and a rating to quantify the relative importance of discussed factors were carried out. The conceptual framework identifies five main components of access to which factors can be assigned: availability, adequacy, acceptability, accessibility and affordability.

Financial factors were considered the most important determinants of access to diagnosis and treatment of tuberculosis. Expenditure for drugs and consultations, for transport, and for special foods as well as lost income were identified as major barriers to treatment. Stigma, doubts about curability and low perceived quality of care were not seen to be significant determinants of access to care for tuberculosis. Community members were well aware of symptoms of tuberculosis and of medical services. These findings were consistent between different respondent groups (community members, patients and providers). They were also highly consistent between the open discussion and the confidential rating.

**Conclusions:**

Illness-costs to households were identified as the main barrier to tuberculosis diagnosis and treatment. To improve access and ultimately adherence to tuberculosis treatment, effective mitigation strategies, e.g. changes in case management, food contributions or financial stimuli, need to be explored and implemented.

## Background

Tuberculosis (TB) cases often delay seeking care even when chemotherapy is free of charge[1,2] and defaulting from treatment is frequent. Irregular drug intake and default can lead to further transmission of TB and drug-resistance[3]. Some of the countries with the highest rates of drug-resistant TB are in Central Asia, making treatment adherence even more important in this region[4]. In order for patients to present timely for diagnosis and for TB cases to be able to adhere to the full treatment, access to care is key. In fact, access has been called "the most important determinant of the outcome of treatment of TB"[5].

In many contexts, TB is linked to stigma and/or perceived to be incurable[6-10]. For those patients believing their illness to have supernatural causes, common sense dictates that they would not utilise biomedical health care. However, in rural Haiti, providing social support had a strong positive influence on adherence, whereas no influence of sorcery beliefs was found[11]. In urban Bolivia, structural barriers including hidden costs of treatment were more important determinants of adherence than socio-cultural factors[12]. TB cases encountered substantial costs already before their diagnosis[13,14], and TB suspects with lower income were less likely to present to a doctor[15]. At large, there are many factors that potentially influence access to care for TB - which ones are the actual determinants is debated.

Tajikistan has seen the breakdown of the previously comprehensive health system. Health care workers are badly paid and poorly motivated. Often patients have to pay for health services that are stipulated to be free of charge. Tajikistan adopted the DOTS strategy in 2002 and reported 79% DOTS coverage, 33% case detection and 84% treatment success in 2006[16,17]. A study found that patients in Tajikistan face average costs of about 4'900USD purchasing power parity (PPP) per TB episode[18]-in a country with a per capita gross domestic product of 1'300USD PPP at the time of the study[19].

The objective of this study was to identify the factors with the strongest influence on access to tuberculosis services - from the point of view of community members, TB patients and health services providers.

## Setting, study population and analytical framework

The present study received ethical approval from the Ministry of Health of Tajikistan. It was conducted in the four pilot districts of Project Sino, which supported the integration of TB services into primary care, funded by the Swiss Agency for Development and Cooperation. DOTS implementation in these districts started in 2004 (Danghara and Varzob) and 2005 (Shahrinaw and Tursunzoda). TB laboratory services and treatment are available at DOTS-centres in each district centre. Primary care facilities are allowed to diagnose TB but virtually always refer suspects to the DOTS-centres for diagnosis[1]. Primary care facilities are requested to provide treatment supervision and follow-up if patients do not present for treatment supervision.

We used a previously described analytical framework of access to care in contexts of livelihood insecurity[20]. Our analysis focussed on access and did no go into details of livelihoods. Availability was not covered, as TB services were known to be available at DOTS-centres. The resulting adapted analytical framework organised factors in four categories: accessibility, affordability, acceptability and adequacy. (Table 1). First, content analysis was conducted. Second, the number of statements relating to each of the four categories were summed up and expressed as a percentage along the three respondent groups. There were hardly any statements relating to adequacy and therefore only the other three categories are presented in the results. Third, results of the rating were analysed by calculating the proportion of chickpeas allocated to each factor, whereby each FGD was equally weighted, independent of the number of participants. The four categories from the content analysis were further divided into twelve sub-categories (Table 1).

**Table 1:** Analytical framework used in this study for factors influencing access to care†

Main categories	Affordability	Acceptability	Accessibility*	Adequacy*
Sub-categories (and examples)	▪ Missed opportunities (lost income due to inability to work)	▪ Side effects of treatment	▪ Distance to facility (effort to cover distance, time needed)	▪ Organisational appropriateness of services
	▪ Medical costs (costs for diagnostic tests, drugs, etc.)	▪ Perceived quality of care (technical specialisation of providers)		▪ Physical state and cleanliness of facilities
	▪ Non-medical costs (transportation costs, expenditure for disease-related diets, etc.)	▪ Attitude of providers (friendliness of doctors)		▪ Convenient opening hours
		▪ Lack of confidentiality (other community members find out about TB patient)		
		▪ Health beliefs (lack of belief in curability)		

†Adapted from reference 20.

*In the participatory approach, factors relating to accessibility and adequacy were not regarded as important (no chickpeas allocated) and the results are presented without these factors.

## Focus Group Discussions

Thirteen focus group discussions (FGDs) were conducted in 2006 to investigate factors influencing access to care for tuberculosis. Five FGDs were conducted with community members, four with TB patients and four with health services providers to cover experiences of stakeholders involved in TB treatment. Stratification along different criteria was used to obtain more valid results (Table 2). Participants were invited by the research team, ensuring different ages were represented. Community members were invited ad hoc, patients were identified through the TB patient registry and health services providers were identified through staff lists. Information on treatment adherence was incomplete. At the time of invitation, patients were asked whether they had been forced to interrupt treatment at any time. Patients reporting irregular treatment intake were included in the same FGD as defaulters. The research team consisted of a moderator, a transcriber and two observers. The moderator with a background in chemistry, community development and applied sociologic research had previous experience with FGDs and an outstanding ability to gain people's trust and respect.

**Table 2:** Eligibility criteria and stratification for the 13 focus group discussions, by respondent category

Community members	Patients	Providers
1) Women from small rural town	1) Women in treatment	1) Family doctors from Danghara
2) Men from remote rural village	2) Men in treatment	2) Nurses from Varzob
3) Women from rural village	3) Women and men after treatment success	3) Family doctors from Shahrinaw and Tursunzoda
4) Men from rural village	4) Defaulters and patients indicating irregular drug intake	4) TB specialists from all four districts
5) Women from semi-urban centre		

The FGDs covered the following topics: (i) symptoms and causes of TB, (ii) perceived appropriateness of treatment, (iii) factors determining the communities' ability to receive diagnosis and (iv) to complete treatment for TB. At the beginning of the FGDs with community members no hint to TB was given. Participants were asked about diseases causing cough; subsequently introducing other symptoms like sputum production, fatigue, weight loss, and night sweats. After the discussion on diseases causing the named symptoms, the focus on TB was disclosed to the participants. In FGDs with patients and providers, the focus on TB was known from the beginning.

After the discussion, participants of community and patient FGDs were asked to rank the factors preventing "people from receiving full medical treatment" by importance in a participatory approach ('rating'), using chickpeas to rank the perceived importance of different factors. No rating was conducted among providers, because it interfered with local concepts of professionalism. For the rating, all factors named during the discussion on access to care were listed. The researchers suggested the following potential factors whenever they were not spontaneously mentioned: attitude of providers, insufficient confidentiality of medical services, stigma of TB, attitude of other community members towards TB patients and lost income. Each participant was asked to confidentially allocate six, three and one chickpea to the three most important factors.

FGDs were conducted in Tajik. Translation to the minority languages Uzbek and Russian were provided by the moderator and an observer as needed. All FGDs were recorded on audiotape and transcribed to English. The accuracy of the transcriptions was checked by the main researcher who is fluent in Tajik and English. Where needed, the whole team re-checked the transcription by listening to the audiotape and by checking their own notes taken during the FGDs. The main researcher read the transcriptions repeatedly, categorised the statements using the analytical framework described above and added codes in TAMS Analyzer (version 3.31b2pt, Matthew Weinstein, 2005).

## Study participants

Fourty-three community members participated in the community FGDs, 21 women and 22 men with a mean age of 42 years. Among providers, 15 doctors, nine nurses and seven TB specialists participated (mean age 45 years). Of 23 patients, eight were women and 15 were men. Participants were mainly from rural areas with about one fourth coming from semi-urban areas.

First the results from the discussion on characteristics of TB and on barriers to treatment are presented and then the relative importance of identified factors from the rating. The topics that came up in the discussion on access to diagnosis and on access to treatment were very much the same. Participants also explicitly stated that the same factors were important at both times. Consequently the results are presented jointly.

## Knowledge about TB

Upon presentation of symptoms of TB, community members mentioned a number of respiratory diseases, most prominently asthma, influenza and bronchitis. Community members also mentioned TB soon in all FGDs. Overall, knowledge of symptoms was accurate. Community members were able to identify medical facilities providing TB treatment in their surroundings.

Knowledge about causes of TB was limited. In particular the belief that TB was inherited and normally incurable was found commonly among community members and also expressed by one patient and one provider. These beliefs were closely linked to the stigmatisation of the illness. The stigma was magnified by the fact that TB services are offered in separate facilities.

Despite the misunderstandings on causes of TB and prospects of care, all respondent groups deemed medical treatment necessary and community members stated that they would seek medical care in case of illness. Community members showed themselves well aware of the availability of TB services at the DOTS centres. Overall it was revealed that due to the difficulties associated with accessing medical care, patients tended to delay presenting until their health status had become severe - despite community members being aware of better prospects of cure at earlier presentation to care.

## Factors determining access to care

Respondents in all FGDs agreed that financial factors were the strongest determinants of access to care and the patient's ability to take the full treatment, i.e. adherence. On the question, why people with TB do not get treatment, a woman (teacher, 47 years old) from Varzob town said: "No money, no treatment - money plays a great role." A general practitioner (40 years) from Danghara answered: "First, because of the financial situation, because of the funds." Other possible reasons were not perceived to be equally important. Although stigma of TB was acknowledged, most participants emphasized it would not prevent patients from visiting medical facilities. Side-effects of treatment were experienced by some participating patients, but were not mentioned very often and were considered important by a small minority only.

**Figure 1:**
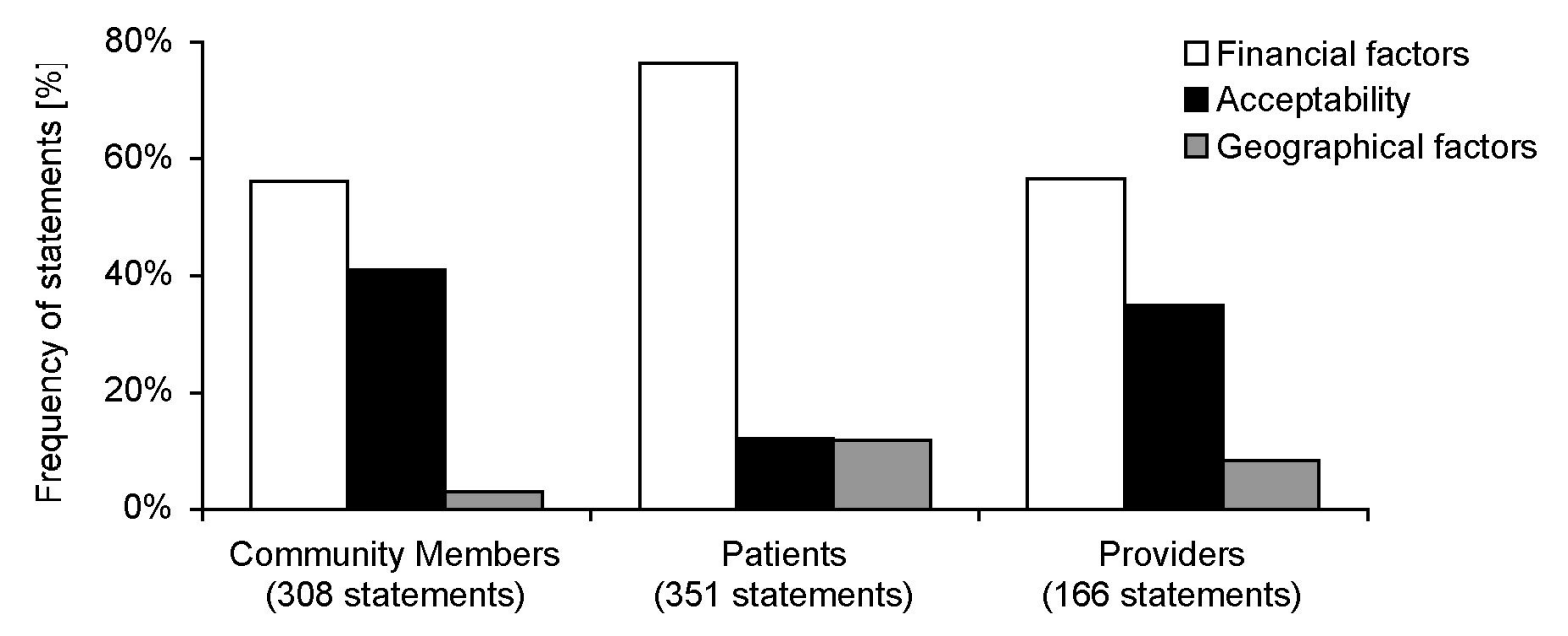
**Frequency of statements referring to three components of access to care**.

The perceived higher importance of economic factors relative to acceptability and accessibility of services was also reflected in the number of statements among all respondent groups (Figure 1). Factors relating to adequacy of services were mentioned even less often and are not presented here. Notably, factors relating to acceptability of services were mentioned least often among patients. Financial factors included all direct costs and lost income. Geographical factors included the effort for the patient to travel and time spent travelling. It did not include transport costs and statements linking travel time to money.

**Figure 2:**
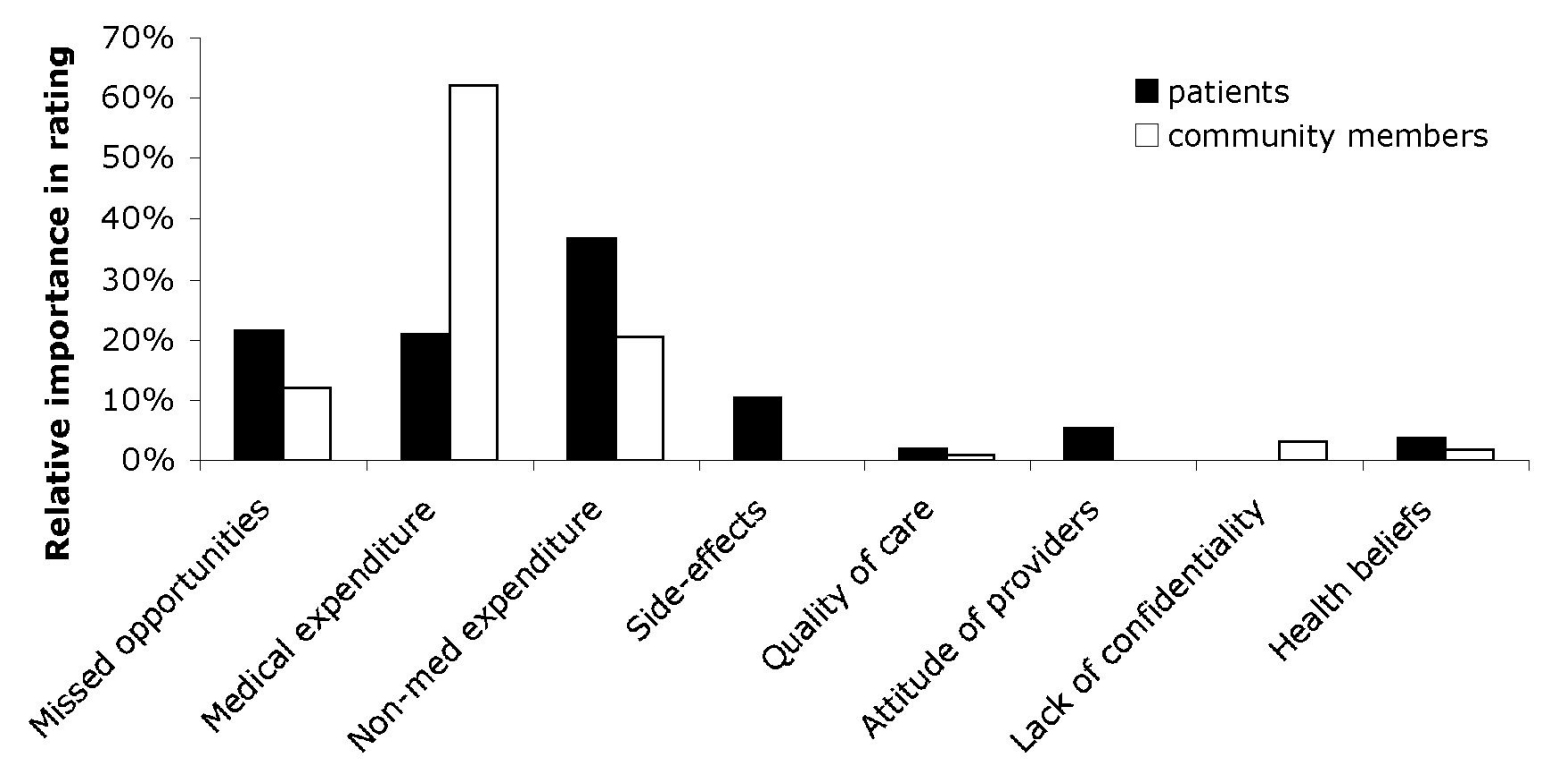
**Relative importance of different barriers to tuberculosis treatment, measured by the proportion of chickpeas allocated by participants of FGDs**.

Community members allocated 12%, 62% and 20% of chickpeas to the categories 'missed opportunities', 'medical expenditure' and 'non-medical expenditure' respectively (Figure 2). Patients allocated 21%, 21% and 37% of chickpeas to the same factors. Thus a total of 94% and 78% of chickpeas respectively were allocated to financial factors. Factors related to acceptability unified only 11% and 6% of chickpeas in patient and community FGDs, respectively, despite the moderator carefully probing for and explaining rational ways of action of these factors.

## Factors contributing to costs

**Figure 3:**
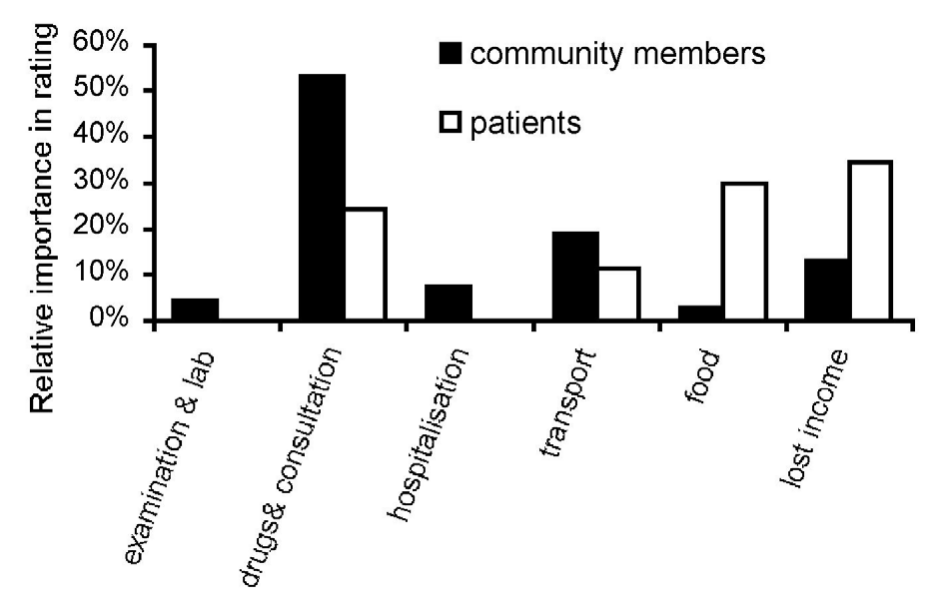
**Relative importance of financial factors regarding access to TB services, measured by the proportion of chickpeas allocated by participants of FGDs**.

Costs mentioned in the FGDs can be grouped into six categories: diagnosis, drugs and consultation, hospitalisation, transport, increased expenditure for food and lost income. Diagnostic expenditure was seen to be less important with the exception of radiography, which was considered expensive by patients and community members. Cost of drugs and consultation were identified as important barriers by patients and especially by community members (Figure 3). Three patients reported they were asked to pay for the TB drugs. For the majority of patients, 'cost of drugs' stands for additional, symptomatic treatment, like vitamin injections or intravenous rehydration. Admission to hospital has received little attention, probably because there is no admission fee. Admission is however linked to expenditure for transport and increased expenditure for food. Food for hospitalised patients has to be provided by caregivers such as family members leading to higher food-related expenditure during hospitalisation. Participants of the FGDs did not always conceptually separate the costs of travel for food provision from the cost of the food itself.

## Significance and implications

While several studies showed that patients face substantial costs even where TB drugs are stipulated to be free of charge[18,21-24], few previous studies have investigated the relative importance of financial versus other barriers to TB care. We investigated the influence of various health system and household-related factors on access to care for TB in Tajikistan. Community members, patients and providers consistently reported that illness costs to households determine access to diagnosis and treatment to a large extent and deemed quality of care, stigma and other factors less important. To our knowledge, this is the first study to demonstrate that illness-related costs are experienced by a broad range of stakeholders including health care providers as the major determinant of access to TB treatment. It is noteworthy that participants named the same factors as barriers to diagnosis and to treatment. Moreover, they stated that the same factors were important in both situations.

The main limitation of this study is possible reporting bias. Reporting bias, however, should be reduced in the confidential participatory approach. The high consistency between the open discussion and the participatory approach lead us to believe that reporting bias was limited. At the time of study implementation, DOTS was relatively new to two of the study districts. Further improvements may have happened since then.

The predominance of financial factors corresponds well to the results of the small number of previous studies investigating the importance of financial and a wide range of other factors[11,12]. The importance of financial factors is amplified for community members by their lack of awareness about anti-mycobacterial therapy being offered free of charge. This is likely to influence their health care seeking.

Enabling patients to obtain prompt diagnosis and full treatment and thus interrupting the cycle of infection is crucial. The results of this study suggest that doing so requires a reduction of the costs faced by patients and their households.

When designing strategies to reduce costs to patients, it should be kept in mind, that components of access to care are interrelated. For example, improvements in the quality and reliability of food provided at the hospital would reduce the need for patients' relatives to travel on a daily basis to bring meals to hospitalised patients. Case management factors, like the provision of additional treatment besides the TB-medication, have been found to cause substantial expenditures for TB patients in Tajikistan[18]. Reviewing the rationality of such additional medication may be needed. For those cases, where the latter is medically warranted, programs and donors should aim at providing additional medication for free, too. The highest costs, however, were related to loss of income due to TB disease[18]. Social support mechanisms including financial stimuli or-as is already being done in Tajikistan-food complements are necessary and may need to be expanded[11,25-27]. Insurance schemes or formalised community support are options to ease the financial hardship of disease, but need to be carefully explored and designed[28]. In the longer term and in contexts with good levels of accountability and governance, such insurance schemes could potentially contribute to improved adherence to TB treatment. Longer delays to TB diagnosis are also associated with higher costs[22,29]. Shortening delays would contribute to lower expenditure as well as a reduction of the loss of income associated with disease.

Concluding, it is difficult to increase TB case detection and treatment adherence as long as patients' immediate concerns are not addressed. For TB control to be successful in Tajikistan and many other countries, programs need to reduce financial barriers to TB treatment. Possible ways to ease the financial hardship of TB for affected households include among others changes in case management, financial stimuli and food supplements.

## Competing interests

The authors declare that they have no competing interests.

## Authors' contributions

RA was the main responsible researcher for all parts of the study. KW contributed to designing the study and to writing the manuscript. HA and SS participated in design of the study and data collection. All authors read and approved the final manuscript.
